# Rhizosphere Bacterial Community Structure and Functional Characteristics Associated with *Fusarium* Wilt Resistance in Banana Germplasms

**DOI:** 10.3390/biology15141186

**Published:** 2026-07-18

**Authors:** Tianyan Yang, Songheng Yi, Qihang Cai, Ziai Zhao, Zhencheng Meng, Jiazeng Zhi, Jianchun Zhang

**Affiliations:** 1 Honghe Tropical Agricultural Institute of Yunnan, Mengzi 661100, China; ytyanyn@163.com (T.Y.); 18314130085@163.com (S.Y.); zhaoziai146@163.com (Z.Z.); mengzhencheng010@163.com (Z.M.); 2 College of Landscape and Horticulture, Southwest Forestry University, Kunming 650224, China; caiqihang98@163.com

**Keywords:** *Fusarium* wilt, banana germplasms, rhizosphere bacterial communities, bacterial community composition, co-occurrence network

## Abstract

The rhizosphere bacterial community is an important determinant of plant health and disease resistance. To investigate bacterial community characteristics associated with *Fusarium* wilt resistance, we used full-length 16S rRNA amplicon sequencing to characterize rhizosphere bacterial communities. The analysis included seven banana germplasms with contrasting resistance levels. Significant genotype-dependent differences in bacterial community composition were detected among the germplasms. Alpha diversity analysis showed that bacterial richness and diversity were not consistently associated with *Fusarium* wilt resistance. In contrast, highly resistant germplasms were enriched in potentially beneficial taxa, including *Bacillus* and *Nitrospira*, whereas *Chujaibacter* and *Acinetobacter* were more abundant in moderately resistant and low-resistant germplasms. Beta diversity analysis further revealed distinct community structures across the germplasms. Functional prediction indicated that bacterial communities associated with highly resistant germplasms possessed greater potential for carbohydrate metabolism, secondary metabolite biosynthesis, and antibiotic biosynthesis. Co-occurrence network analysis revealed distinct bacterial interaction patterns among germplasms with different resistance levels. Highly resistant germplasms showed strong associations among several key bacterial taxa. In contrast, moderately resistant and low-resistant germplasms exhibited different patterns of positive and negative bacterial associations. Collectively, these results suggest that *Fusarium* wilt resistance in banana is associated not with increased bacterial diversity but with the enrichment of potentially beneficial bacterial taxa, enhanced bacterial functional potential, and distinct bacterial interaction patterns.

## Simple Summary

Banana *Fusarium* wilt is one of the most serious diseases affecting banana production worldwide and remains difficult to control. Bacteria living in the soil surrounding plant roots play important roles in plant health and may help plants resist disease. In this study, we compared the root-associated bacterial communities of seven banana germplasms with different levels of resistance to *Fusarium* wilt. We found that each germplasm harbored a distinct bacterial community. Highly resistant germplasms tended to contain higher abundances of potentially beneficial bacteria and exhibited distinct interaction patterns involving several key bacterial groups. In contrast, bacterial diversity alone was not consistently related to disease resistance. These results suggest that resistance to *Fusarium* wilt is associated with differences in root-associated bacterial community composition, functional potential, and bacterial interaction patterns rather than simply the number of bacterial species present. This study improves our understanding of how root-associated bacteria may contribute to disease resistance in banana. It also provides useful information for developing environmentally sustainable strategies to manage *Fusarium* wilt.

## 1. Introduction

Banana (*Musa* spp.) is an important food and cash crop cultivated in more than 130 countries and provides food and income for over 400 million people worldwide. It is also a major agricultural commodity in many tropical and subtropical regions [1]. However, *Fusarium* wilt (Panama disease), caused by *Fusarium oxysporum* f. sp. *cubense* (Foc), is one of the most destructive soil-borne diseases threatening banana production globally [2]. The pathogen infects the vascular system of banana plants, resulting in leaf yellowing, pseudostem discoloration, wilting, and eventual plant death. Its capacity to survive in soil for prolonged periods as spores contributes to its widespread occurrence and complicates disease management [3]. Historically, *Fusarium* wilt has caused severe losses in several major commercial banana cultivars and has substantially altered the global banana industry [2]. In recent decades, the spread of different Foc races, particularly Tropical Race 4 (Foc TR4), has expanded *Fusarium* wilt into major banana-producing regions of Asia, Africa, the Middle East, and South America. This global spread poses a persistent threat to banana production and food security in tropical and subtropical regions [4,5,6]. Because most cultivated banana varieties exhibit limited resistance and available control measures provide inconsistent results, the long-term management of *Fusarium* wilt remains a major challenge [7,8].

Current management of banana *Fusarium* wilt relies primarily on agricultural practices, including quarantine measures, soil treatment, eradication of infected plants, and chemical control. However, their effectiveness remains limited. Single-site fungicides generally show poor efficacy against *Fusarium* wilt because Foc can develop reduced sensitivity to these compounds [8,9]. Although multi-site fungicides can suppress disease development under field conditions, their performance is often inconsistent and strongly influenced by environmental factors, limiting their long-term effectiveness [8]. Strict quarantine measures and the use of pathogen-free planting materials are important strategies for preventing pathogen spread, but their implementation is challenging in already infested soils. In addition, soil fumigation, liming, and crop rotation may reduce pathogen populations. However, their effects are often temporary because Foc chlamydospores can survive in soil for extended periods [3,7]. Consequently, existing chemical and physical control strategies are insufficient to provide sustainable control of banana *Fusarium* wilt.

Unlike conventional chemical and physical control strategies, biological control mediated by rhizosphere microorganism targets *Fusarium* wilt through ecological processes within the rhizosphere. Its effectiveness is largely attributed to the establishment of specific microbial community structures and functions that restrict Foc colonization and infection [10]. Beneficial microorganisms can suppress pathogen growth and infection through multiple mechanisms, including the production of antimicrobial metabolites, secretion of cell wall-degrading enzymes, competition for limiting nutrients such as iron, and induction of systemic resistance in host plants [11,12]. These activities can also influence rhizosphere community composition and microbial interaction networks, contributing to the development of disease-suppressive soils [13,14]. Numerous biocontrol microorganisms isolated from disease-suppressive soils and the rhizosphere of healthy banana plants, including *Bacillus*, *Pseudomonas*, *Streptomyces*, and *Trichoderma*, exhibit strong antagonistic activity against Foc. These microorganisms can reduce disease incidence through the production of antimicrobial compounds, secretion of cell wall-degrading enzymes, and promotion of plant growth [12,15,16,17]. Community-level studies have shown that healthy soils are often enriched in beneficial microbial taxa, whereas diseased soils tend to contain higher proportions of pathogenic fungi and less complex microbial networks, particularly in long-term monoculture systems [18]. Inoculation with beneficial microorganisms can partially restore rhizosphere microbial diversity and reconstruct disease-suppressive community structures, thereby reducing the incidence of *Fusarium* wilt [18,19,20]. Furthermore, increasing evidence indicates that plant genotypes can selectively recruit specific rhizosphere microorganisms. Disease-resistant genotypes tend to enrich taxa with antagonistic or beneficial functions. This highlights the potential contribution of host-mediated microbiome assembly to disease resistance [21].

*Fusarium* wilt remains a major constraint on banana production, highlighting the need for effective and sustainable management strategies. Recent studies on the banana rhizosphere microbiota have focused primarily on the identification of beneficial microorganisms, elucidation of biocontrol mechanisms, and characterization of rhizosphere community dynamics. Although numerous microbial taxa associated with disease suppression have been reported, most studies have focused on individual biocontrol strains or single resistant cultivars. Consequently, systematic comparisons of rhizosphere bacterial communities among banana germplasms with different levels of *Fusarium* wilt resistance remain limited. In particular, the relationships between bacterial community composition, functional potential, and host resistance have not been comprehensively evaluated at the community level. This study aimed to investigate the rhizosphere bacterial community characteristics associated with *Fusarium* wilt resistance in banana germplasms. Rhizosphere bacterial communities are key microbial components associated with soil-borne disease suppression and plant resistance [22]. Therefore, seven banana germplasms exhibiting contrasting levels of *Fusarium* wilt resistance were selected to characterize bacterial community composition and functional potential associated with resistance variation. Using full-length 16S rRNA amplicon sequencing, we characterized their rhizosphere bacterial communities and compared bacterial diversity, community composition, functional potential, and co-occurrence network characteristics across germplasms with different resistance levels. This study provides insights into the bacterial community characteristics associated with *Fusarium* wilt resistance and contributes to the identification of microbial resources with potential applications in biological control.

## 2. Materials and Methods

### 2.1. Banana Germplasms with Different Levels of Fusarium Wilt Resistance

**Figure 1:**
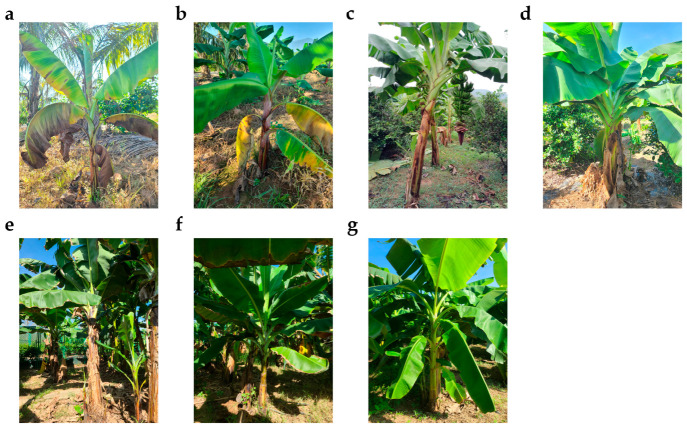
Banana germplasms with different levels of resistance to *Fusarium* wilt. (**a**) Dianjiao No. 1 (DJ1H); (**b**) Hongyan No. 5 (HY5H); (**c**) Dianjiao No. 3 (DJ3H); (**d**) Dianjiao No. 4 (DJ4H); (**e**) Nantianhuang (NTH); (**f**) Baodaojiao (BDJ); (**g**) Dajiao (DJ).

The banana germplasms used in this study (*Musa acuminata*, AAA Group, Cavendish Subgroup) were cultivated at the Provincial Germplasm Repository of Characteristic Tropical Fruit Trees, Honghe Tropical Agricultural Science Research Institute, Hekou County, Yunnan Province, China (103°52′43.32″ E, 22°34′2.77″ N). Based on their resistance to *Fusarium* wilt, Dianjiao No. 1 (DJ1H) was classified as a low-resistant germplasm; Hongyan No. 5 (HY5H) and Dianjiao No. 3 (DJ3H) were classified as moderately resistant germplasms; and Dianjiao No. 4 (DJ4H), Nantianhuang (NTH), Baodaojiao (BDJ), and Dajiao (DJ) were classified as highly resistant germplasms (Figure 1). BDJ and NTH have been previously reported as highly resistant germplasms to *Fusarium* wilt [23,24,25]. The resistance classifications of the remaining germplasms were based on previous disease resistance evaluations conducted by our research team [26]. The corresponding disease severity indices and resistance classifications of all seven germplasms are summarized in Table S1. The seven germplasms were selected to cover a resistance gradient from low to high within a genetically comparable *Musa acuminata* (AAA, Cavendish subgroup) background, based on availability and prior resistance records.

### 2.2. Sample Collection

The sampled plants were healthy banana plants at the booting stage (mature growth stage) with no visible symptoms of disease or insect damage and similar growth status, randomly selected from the germplasm repository. The soil type at the experimental site was sandy loam. The soil exhibited a pH of 6.42, with an organic matter content of 11.8303 g kg^−1^. The contents of total nitrogen, total phosphorus, and total potassium were 0.8049, 0.2003, and 13.3992 g kg^−1^, respectively. The available potassium content was 229.0423 mg kg^−1^, and the available concentrations of Cu, Fe, Mn, and Zn were 2.2183, 31.7539, 20.6175, and 1.2334 mg kg^−1^, respectively. Rhizosphere soil samples were collected under aseptic conditions. Entire root systems were carefully excavated, and loosely attached bulk soil was removed by gentle shaking. Plant debris, stones, and other impurities adhering to the root surface were then re-moved. Rhizosphere soil tightly attached to the roots was collected using a sterile brush.

For each germplasm, rhizosphere soil samples were collected using a five-point sam-pling method. Samples were placed in sterile sealed bags immediately after collection and transported to the laboratory under refrigerated conditions. All samples were flash-frozen in liquid nitrogen and stored at −80 °C until further use. Samples were subsequently transported on dry ice for downstream sequencing analyses.

### 2.3. Full-Length 16S rRNA Gene Sequencing of Rhizosphere Bacterial Communities

Total microbial DNA was extracted from each soil sample using the TianGen DNA extraction kit (TianGen Ltd., Beijing, China). DNA quality was evaluated by agarose gel electrophoresis and spectrophotometry. DNA samples with OD_260_/_280_ values between 1.8 and 2.0 were selected and stored at −20 °C until further use. The full-length bacterial 16S rRNA gene was amplified using the forward primer F: AGRGTTTGATYNTGGCTCAG and the reverse primer R: TASGGHTACCTTGTTASGACTT. PCR products that met quality requirements were used for library construction and sequencing. Amplicons were purified and used to construct SMRTbell libraries. Qualified libraries were sequenced on the PacBio Sequel II platform using single-molecule real-time (SMRT) sequencing. Circular consensus sequencing (CCS) reads were generated using SMRT Link software v13.1. Raw sequencing data were subsequently processed using lima v1.7.0, cutadapt v1.9.1, and UCHIME v4.2 for demultiplexing, adapter trimming, and chimera removal. High-quality CCS reads were retained for downstream analyses. The raw sequencing reads generated in this study were deposited in the NCBI Sequence Read Archive (SRA) database under accession number PRJNA1492761.

### 2.4. OTU Clustering and Taxonomic Annotation

High-quality CCS reads were clustered into operational taxonomic units (OTUs) using USEARCH at a 97.0% sequence similarity threshold. Considering the high accuracy and full-length coverage of PacBio CCS reads, OTU-based clustering was retained in this study for bacterial community profiling, which has been widely applied in full-length 16S rRNA gene sequencing analyses [27]. OTUs with fewer than three reads were removed. Taxonomic classification was performed against the SILVA 138 reference database. Representative OTU sequences were assigned to seven taxonomic levels, including kingdom, phylum, class, order, family, genus, and species, using a combination of BLAST v.2.13.0 and Bayesian classification methods. The abundance and richness of taxa at different taxonomic levels were subsequently calculated for each sample to characterize bacterial community composition.

### 2.5. Bioinformatics Analysis

Based on the taxonomic annotation results, bacterial community diversity analyses were performed using QIIME2 (v2020.6). Alpha diversity indices were compared among groups using one-way analysis of variance (ANOVA) followed by Tukey’s multiple comparison test at *p* < 0.05. Differential taxa among groups were identified using ANOVA, rank-sum tests, Metastats, and STAMP-based statistical analyses. Core biomarkers associated with the rhizosphere bacterial communities of banana germplasms exhibiting different levels of *Fusarium* wilt resistance were identified using linear discriminant analysis effect size (LEfSe) with an LDA score > 4.0 and *p* < 0.05. At the genus level, co-occurrence networks of rhizosphere bacteria were constructed based on Spearman’s rank correlation analysis using thresholds of |*r*| > 0.5 and *p* < 0.01 [28,29]. Functional profiles of bacterial communities were predicted using PICRUSt2 based on the KEGG and COG databases. Differences in predicted functions among groups were analyzed using STAMP software v2.1.3.

## 3. Results

### 3.1. Quality Assessment of 16S rRNA Sequencing and OTU Analysis

**Figure 2:**
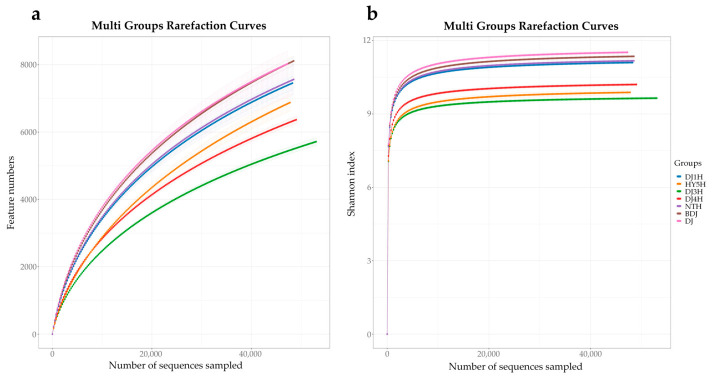
Rarefaction analysis of rhizosphere bacterial communities in different banana germplasms. (**a**) Rarefaction curves; (**b**) Shannon diversity rarefaction curves.

**Figure 3:**
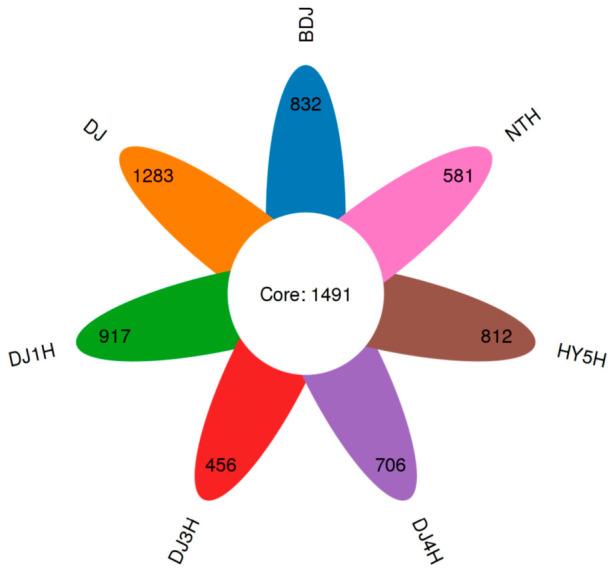
Petal diagram showing shared and unique OTUs among rhizosphere bacterial communities of different banana germplasms.

A total of 28 rhizosphere soil samples from seven banana germplasms exhibiting different levels of *Fusarium* wilt resistance were subjected to full-length 16S rRNA gene sequencing. The number of raw CCS reads per sample ranged from 53,814 to 68,188. After removal of redundant sequences and length filtering, 50,922–64,919 high-quality CCS reads were retained for subsequent analyses. The lengths of the effective sequences ranged from 1447 to 1456 bp across all samples, and the proportion of effective sequences varied from 89.36% (HY5H-2) to 98.10% (DJ3H-3), indicating that the sequencing data were of sufficient quality for downstream analyses (Table S2). Both the rarefaction and Shannon diversity curves approached saturation, suggesting that the sequencing depth was sufficient to capture most of the bacterial diversity present in the samples (Figure 2). OTU analysis identified 1491 OTUs shared among all banana germplasms, representing the core bacterial taxa shared among all germplasms (Figure 3). Among these core OTUs (Table S3), Proteobacteria was the predominant phylum, followed by Firmicutes, Bacteroidota, Actinobacteriota, and Nitrospirota. At the genus level, *Nitrospira*, *Bacillus*, *Sphingomonas*, *Pseudolabrys*, and *Gaiella* were among the dominant taxa within the core bacterial community. These results indicate that banana germplasms shared a conserved core rhizosphere bacterial community, which was mainly composed of dominant bacterial lineages commonly detected in agricultural soils. In addition, each germplasm contained a number of unique OTUs. Among them, the highly resistant germplasm DJ had the greatest number of unique OTUs (1283), followed by the low-resistant germplasm DJ1H (917), whereas the moderately resistant germplasm DJ3H had the fewest unique OTUs (456). These results indicate substantial differences in OTU distribution among banana germplasms.

### 3.2. Analysis of Rhizosphere Bacterial Community Composition

**Figure 4:**
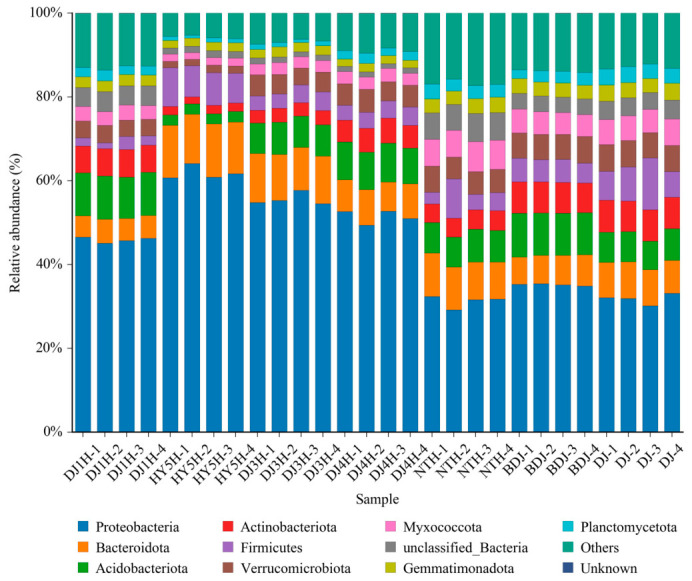
Relative abundance of rhizosphere bacterial communities at the phylum level across different banana germplasms.

**Figure 5:**
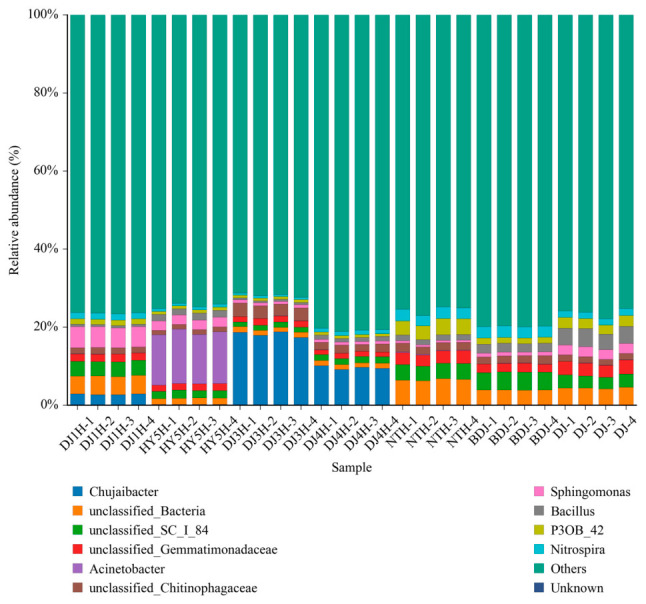
Relative abundance of rhizosphere bacterial communities at the genus level across different banana germplasms.

Taxonomic annotation identified 37 phyla, 92 classes, 307 orders, 681 families, 1589 genera, and 4283 species-level taxa across all samples (Table S4). At the phylum level, Proteobacteria, Actinobacteriota, Acidobacteriota, Bacteroidota, and Firmicutes were the dominant bacterial phyla and constituted the majority of the rhizosphere bacterial community (Figure 4). At the genus level, the relative abundances of dominant bacterial taxa varied substantially among banana germplasms with different levels of *Fusarium* wilt resistance (Figure 5). *Chujaibacter* exhibited a distinct enrichment pattern. Its relative abundance reached 17.31–18.76% in the moderately resistant germplasm DJ3H and 9.11–10.14% in the highly resistant germplasm DJ4H, whereas its abundance in all other germplasms was below 0.03%. *Acinetobacter* was enriched in the moderately resistant germplasm HY5H, with a relative abundance of 12.63–13.92%, while remaining at very low abundance in the other germplasms. *Sphingomonas* showed the highest relative abundance in the low-resistant germplasm DJ1H (5.08–5.41%), followed by the highly resistant germplasms DJ and BDJ (2.42–2.58%). The lowest abundances were observed in the moderately resistant germplasm DJ3H and the highly resistant germplasms DJ4H and NTH (0.07–0.08%). *Bacillus* was more abundant in the highly resistant germplasms DJ and BDJ, with relative abundances of 3.93–4.61% and 2.15–2.29%, respectively. In comparison, its abundance in the moderately resistant and low-resistant germplasms ranged from 0.05% to 0.07%.

### 3.3. Alpha Diversity Analysis of Rhizosphere Bacterial Communities

**Figure 6:**
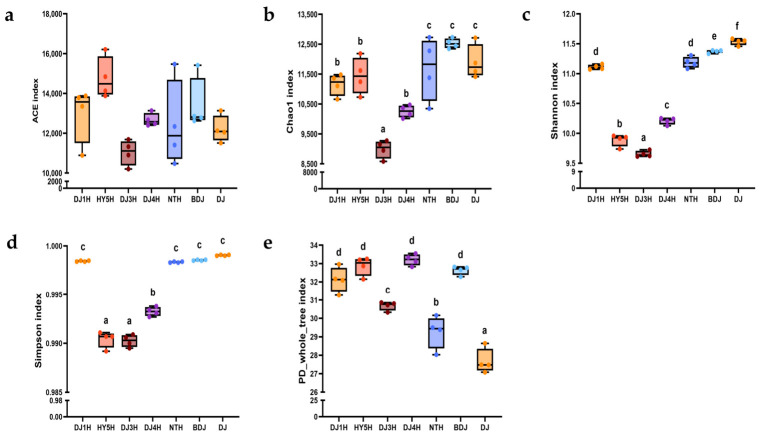
Alpha diversity indices of rhizosphere bacterial communities in banana germplasms with different levels of *Fusarium* wilt resistance. (**a**) ACE index; (**b**) Chao1 index; (**c**) Shannon index; (**d**) Simpson index; (**e**) PD whole tree index. Significant differences among germplasms were determined by one-way analysis of variance (ANOVA) followed by Tukey’s honestly significant difference (HSD) test at *p* < 0.05. Different lowercase letters indicate significant differences among germplasms.

To evaluate differences in rhizosphere bacterial community diversity among banana germplasms, alpha diversity indices were compared across the different germplasms. Significant differences in bacterial richness and diversity were observed among germplasms with different levels of *Fusarium* wilt resistance (Table S5). The ACE and Chao1 indices showed variation in community richness among germplasms. HY5H and BDJ generally exhibited higher richness values. Among them, HY5H showed the highest ACE index, ranging from 13,880.74 to 16,206.10 (Figure 6a), whereas BDJ exhibited Chao1 values ranging from 12,346.38 to 12,728.46 (Figure 6b). NTH and DJ showed intermediate richness levels, while DJ3H consistently exhibited lower richness, with Chao1 values ranging from 8581.31 to 9271.95. The Shannon and Simpson indices indicated differences in community diversity and evenness among germplasms. DJ and BDJ exhibited relatively high diversity, with DJ showing the highest Shannon index (11.46–11.58) and Simpson index values consistently above 0.999 (Figure 6c,d). In contrast, DJ3H and HY5H exhibited comparatively lower Shannon diversity, with DJ3H showing the lowest values (9.61–9.73). The PD whole tree index also varied among germplasms. DJ4H, HY5H, BDJ, and DJ1H exhibited relatively high phylogenetic diversity, with DJ4H showing the highest values (32.83–33.55), whereas DJ and NTH showed comparatively lower values (Figure 6e). Overall, alpha diversity indices differed among banana germplasms, reflecting variation in bacterial richness, diversity, evenness, and phylogenetic diversity across the rhizosphere bacterial communities.

### 3.4. Beta Diversity Analysis of Rhizosphere Bacterial Communities

**Figure 7:**
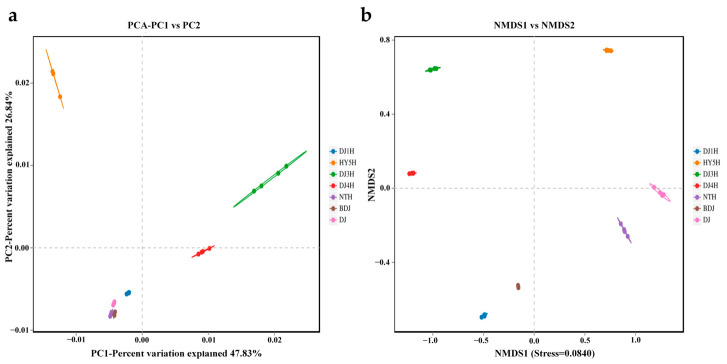
Beta diversity of rhizosphere bacterial communities in banana germplasms with different levels of *Fusarium* wilt resistance. (**a**) PCA plot; (**b**) NMDS plot.

To further evaluate differences in rhizosphere bacterial community composition among banana germplasms, beta diversity analyses were performed. Principal component analysis (PCA) showed that DJ3H and HY5H occupied distinct positions in the ordination space and were clearly separated from the other germplasms. DJ4H was also separated from DJ1H, BDJ, and NTH, indicating differences in bacterial community structure among these germplasms (Figure 7a). The NMDS analysis yielded a stress value of 0.084, indicating a good representation of community variation by the ordination model (Figure 7b). DJ, HY5H, and NTH were mainly distributed in the negative region of the NMDS1 axis and were further separated along the NMDS2 axis. In contrast, DJ1H, BDJ, DJ4H, and DJ3H were primarily distributed in the positive region of the NMDS1 axis and also exhibited separation along the NMDS2 axis. These patterns reflected differences in bacterial community composition among the banana germplasms. ANOSIM based on the Bray–Curtis distance matrix revealed significant differences in rhizosphere bacterial community structure among germplasms (*R* = 0.945, *p* = 0.001), indicating that variation among germplasms exceeded variation within germplasms (Figure S1). Overall, beta diversity analyses demonstrated clear differences in rhizosphere bacterial community structure among the seven banana germplasms.

### 3.5. Analysis of Differential Bacterial Taxa and Key Rhizosphere Bacterial Groups

**Figure 8:**
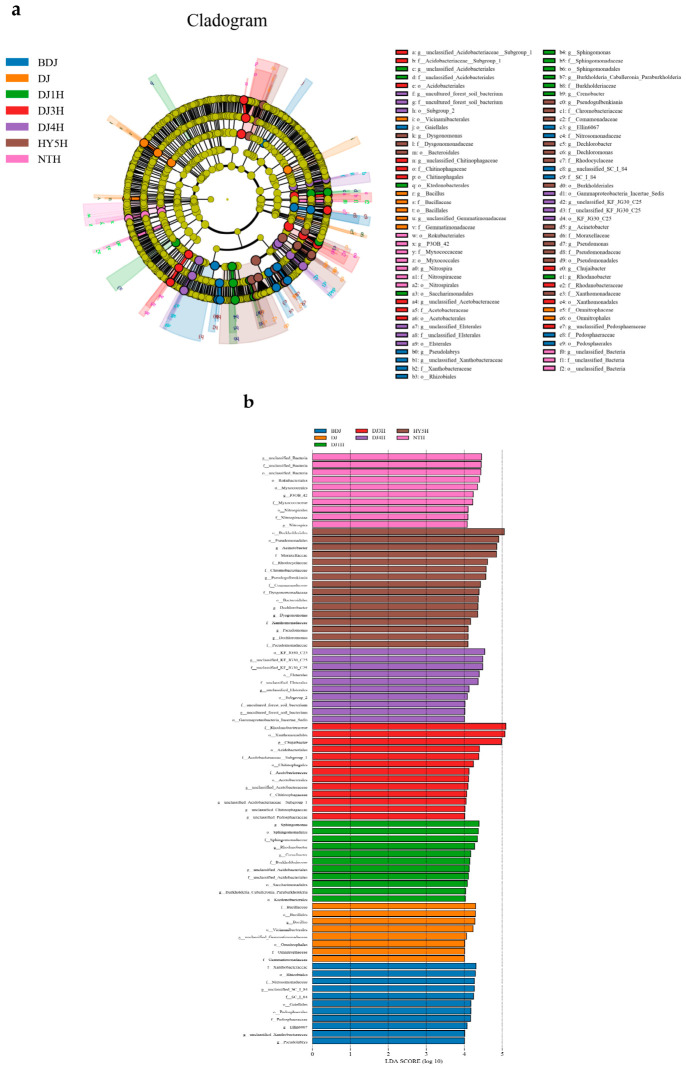
LEfSe analysis of rhizosphere bacterial communities in banana germplasms with different levels of *Fusarium* wilt resistance. (**a**) LEfSe cladogram showing differentially enriched bacterial taxa; (**b**) LDA score distribution of bacterial biomarkers (LDA score > 4.0).

To further characterize differences in rhizosphere bacterial community composition among banana germplasms with different levels of *Fusarium* wilt resistance, differential taxonomic analysis was performed to identify characteristic bacterial taxa. LEfSe analysis revealed distinct bacterial community structures among the seven germplasms, with clear differences in dominant taxa among germplasms exhibiting different resistance levels (Figure 8a). LDA score analysis further identified a series of germplasm-specific biomarker taxa (Figure 8b). The low-resistant germplasm DJ1H was characterized by the enrichment of *Burkholderia*–*Caballeronia*–*Paraburkholderia*, *Sphingomonas*, *Rhodanobacter*, and related taxa. The moderately resistant germplasms DJ3H and HY5H exhibited distinct enrichment patterns. In DJ3H, members of Chitinophagaceae, Acidobacteriales, and Rhodanobacteraceae were significantly enriched. In contrast, HY5H was characterized by the enrichment of *Pseudomonas*, *Acinetobacter*, and taxa affiliated with Comamonadaceae and Rhodocyclaceae. Differences in biomarker taxa were also observed among the highly resistant germplasms. DJ was significantly enriched in *Bacillus* and taxa affiliated with Gemmatimonadaceae. NTH was characterized by the enrichment of *Nitrospira*, Myxococcaceae, and related taxa. BDJ showed enrichment of members of Rhizobiales and Xanthobacteraceae. Although the specific biomarker taxa differed among the highly resistant germplasms, each germplasm exhibited a distinct bacterial enrichment profile.

### 3.6. Functional Prediction Analysis of Rhizosphere Bacterial Communities

**Figure 9:**
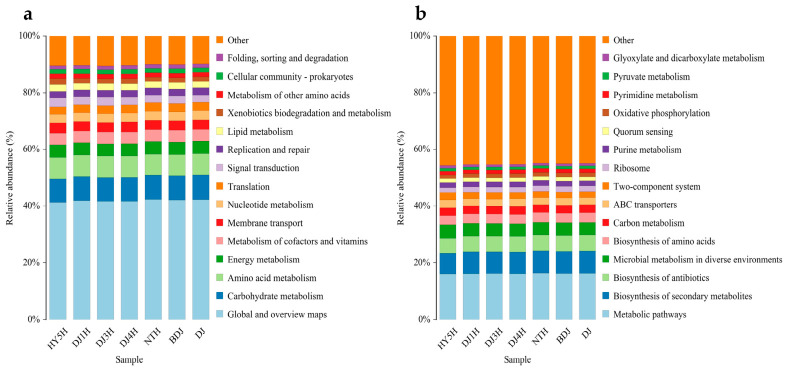
KEGG-based functional prediction of rhizosphere bacterial communities in banana germplasms with different levels of *Fusarium* wilt resistance. (**a**) KEGG Level 2 functional categories; (**b**) KEGG Level 3 functional categories.

To further investigate potential functional differences among rhizosphere bacterial communities associated with banana germplasms exhibiting different levels of *Fusarium* wilt resistance. PICRUSt2 was used to infer microbial functional potential based on 16S rRNA gene profiles and KEGG annotations. The KEGG Level 2 analysis showed that the predicted functional profiles of rhizosphere bacterial communities were dominated by fundamental metabolic categories, including global and overview maps, carbohydrate metabolism, amino acid metabolism, and energy metabolism (Figure 9a). Among the highly resistant germplasms, DJ, BDJ, and NTH were predicted to possess relatively higher abundances of functional pathways associated with carbohydrate metabolism. The corresponding predicted values were 0.0874, 0.0861, and 0.0859, respectively. DJ and NTH were also predicted to have relatively higher abundances of pathways associated with translation and replication and repair. HY5H was predicted to exhibit relatively higher abundances of functional pathways associated with signal transduction (0.0318), membrane transport (0.0362), and xenobiotics biodegradation and metabolism (0.0209). DJ1H was predicted to possess relatively higher abundances of functions associated with lipid metabolism and metabolism of other amino acids. The predicted functional profiles of DJ3H and DJ4H were intermediate between those inferred for the highly resistant and low-resistant germplasms. In addition, DJ4H was predicted to exhibit a relatively higher abundance of functions assigned to the cellular community–prokaryotes category.

The KEGG Level 3 analysis revealed that the predicted functions of rhizosphere bacterial communities were predominantly enriched in pathways related to metabolic pathways, biosynthesis of secondary metabolites, biosynthesis of antibiotics, and carbon metabolism (Figure 9b). Among the highly resistant germplasms, NTH, BDJ, and DJ were predicted to possess relatively higher abundances of functional pathways associated with biosynthesis of secondary metabolites, with predicted values of 0.0771, 0.0765, and 0.0770, respectively. The predicted abundances of functional pathways associated with biosynthesis of antibiotics in these germplasms were 0.0574, 0.0570, and 0.0576, respectively. DJ4H and BDJ were predicted to exhibit relatively higher abundances of functions associated with quorum sensing. HY5H was predicted to exhibit relatively higher abundances of functional pathways associated with microbial metabolism in diverse environments, two-component system, and ABC transporters. Notably, the low-resistant germplasm DJ1H was predicted to possess abundances of functional pathways associated with biosynthesis of secondary metabolites (0.0751) and biosynthesis of antibiotics (0.0564) that were comparable to the predicted values observed in some highly resistant germplasms, despite differences in bacterial community composition among the germplasms.

### 3.7. Co-Occurrence Network Analysis of Rhizosphere Bacterial Communities

**Figure 10:**
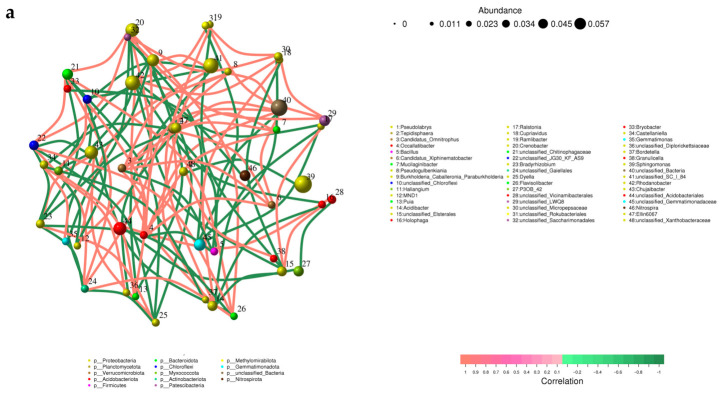

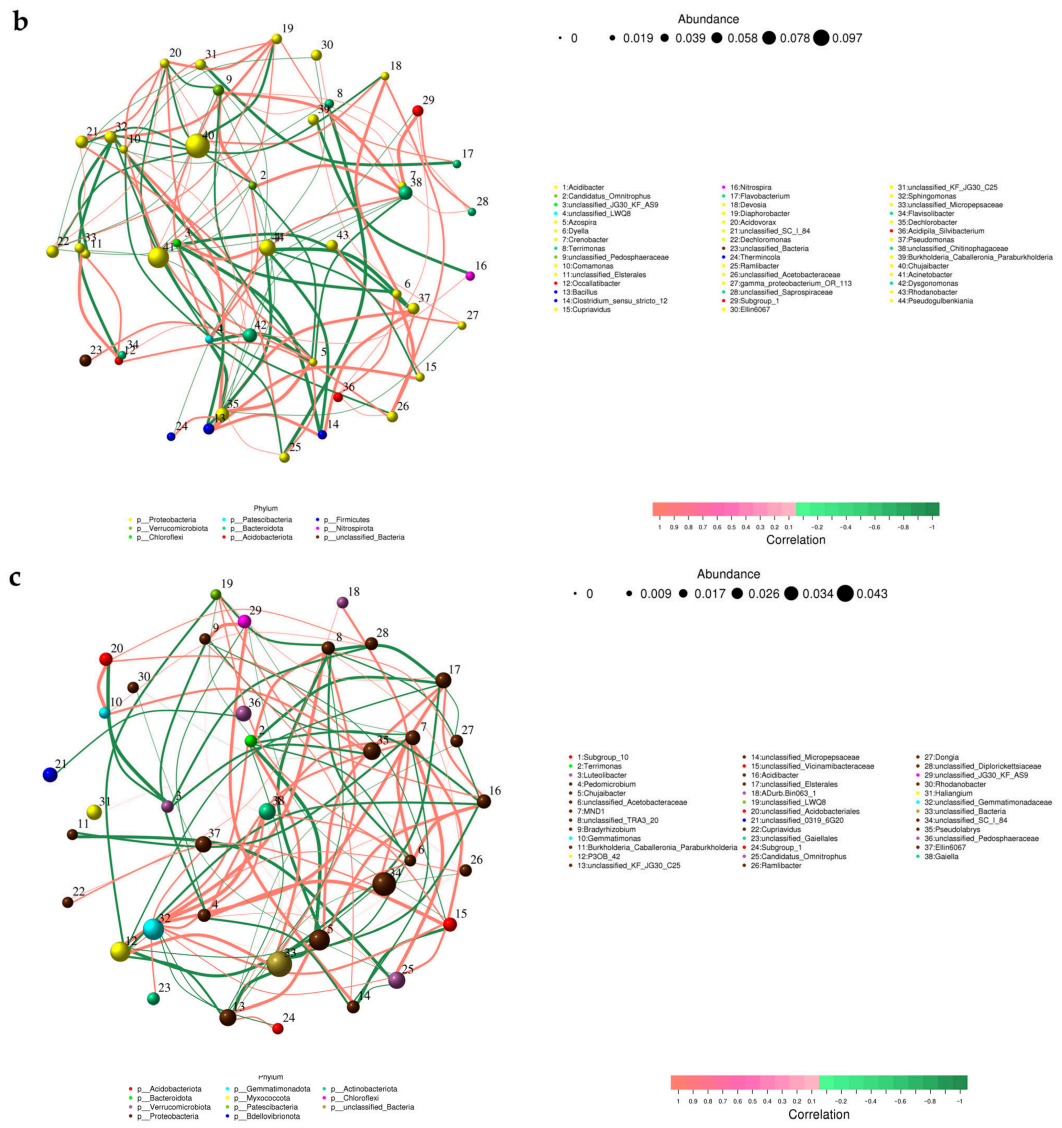
Co-occurrence network of rhizosphere bacterial communities in banana germplasms with different levels of *Fusarium* wilt resistance. (**a**) Low-resistant germplasms; (**b**) Moderately resistant germplasms; (**c**) Highly resistant germplasms.

Co-occurrence networks of rhizosphere bacteria were constructed at the genus level based on dominant bacterial taxa from banana germplasms with different levels of *Fusarium* wilt resistance (Figure 10). The results showed that bacterial communities of all resistance groups formed interaction networks containing both positive and negative correlations. Distinct differences in core bacterial taxa and association patterns were observed among germplasms with different resistance levels. In the low-resistant germplasms (Figure 10a), the network mainly involved *Acidibacter*, *Rhodanobacter*, *Chujaibacter*, *Crenobacter*, and multiple unclassified bacterial taxa. Among them, *Acidibacter* showed a significant positive correlation with *Rhodanobacter* but negative correlations with unclassified_Elsterales, *Holophaga*, and *Ralstonia*. *Chujaibacter* was positively correlated with *Bradyrhizobium* and *Dyella*, while exhibiting negative correlations with *Crenobacter*, unclassified_Chitinophagaceae, unclassified_JG30_KF_AS9, and other bacterial taxa. These results indicated that rhizosphere bacterial communities in low-resistant germplasms exhibited complex cooperative and antagonistic relationships, with diverse association patterns among dominant bacterial taxa. In the moderately resistant germplasms (Figure 10b), *Pseudomonas*, *Bacillus*, *Acidibacter*, *Azospira*, *Dechlorobacter*, and *Chujaibacter* represented major associated taxa within the network. *Pseudomonas* showed significant positive correlations with *Bacillus* and Clostridium_sensu_stricto_12, but a negative correlation with *Acidibacter*. *Azospira* formed significant positive associations with *Dechlorobacter*, *Acidovorax*, and *Comamonas*. Meanwhile, *Chujaibacter* was positively correlated with *Devosia* but negatively correlated with *Dechloromonas*, *Diaphorobacter*, and unclassified_SC_I_84, indicating that bacterial taxa in moderately resistant germplasms formed complex interaction patterns. In the highly resistant germplasms (Figure 10c), *Terrimonas*, *Chujaibacter*, *Acidibacter*, MND1, *Pedomicrobium*, and *Gemmatimonas* were identified as major associated taxa and formed multiple strong cooperative associations. Specifically, MND1 was significantly positively correlated with Candidatus_Omnitrophus, *Gemmatimonas* was positively correlated with unclassified_Acidobacteriales, and *Pedomicrobium* showed significant positive correlations with MND1. In addition, *Terrimonas* exhibited positive correlations with P3OB_42 and unclassified_TRA3_20, while *Chujaibacter* was positively correlated with *Acidibacter* and unclassified_Acetobacteraceae. These results suggested that several dominant bacterial taxa in highly resistant germplasms formed strong cooperative association structures, which might contribute to maintaining specific rhizosphere microbial functions. Overall, rhizosphere bacterial communities of banana germplasms with different resistance levels differed not only in dominant bacterial taxa but also in bacterial interaction patterns. Highly resistant germplasms exhibited stronger cooperative associations among several key bacterial taxa, whereas low- and moderately resistant germplasms showed more complex patterns involving both positive and negative correlations. These findings suggested that *Fusarium* wilt resistance in banana might be closely associated with the restructuring of rhizosphere microbial community organization and bacterial interaction relationships.

## 4. Discussion

*Fusarium* wilt is one of the most destructive soil-borne diseases affecting banana production worldwide. Regulation of rhizosphere bacterial communities has been considered a promising strategy for sustainable disease management [30]. Beneficial bacteria can suppress pathogen infection through nutrient competition, production of antimicrobial compounds, and induction of host resistance [19]. Consequently, the composition and functional attributes of rhizosphere bacterial communities are closely associated with plant health and disease resistance [13]. Host genotype is an important factor shaping rhizosphere bacterial community composition. Different banana germplasms can selectively recruit specific bacterial taxa through differences in root exudation patterns, nutrient utilization strategies, and host traits, resulting in genotype-dependent bacterial community assemblages [17,30,31]. Consistent with previous studies, clear differences in rhizosphere bacterial community composition and structure were observed among the seven banana germplasms. This suggests that host genotype has a strong influence on bacterial community assembly. A relatively high proportion of unclassified bacterial taxa was observed across all samples. This likely reflects the presence of phylogenetically diverse and deeply branching microbial lineages commonly found in tropical and subtropical soils, where taxonomic resolution remains limited for some rhizosphere-associated microorganisms. Previous studies have shown that increased microbial diversity is not always associated with enhanced disease resistance [32,33]. Similarly, the alpha diversity indices observed in this study did not exhibit a consistent relationship with *Fusarium* wilt resistance. These results suggest that *Fusarium* wilt resistance among banana germplasms is more closely associated with specific bacterial taxa and overall community structure than with bacterial richness or diversity alone.

Differences in rhizosphere bacterial community composition among plant genotypes have been widely reported, and specific bacterial taxa are often associated with disease resistance [34]. For example, enrichment of *Streptomyces* has been observed in the rhizosphere of disease-resistant tomato plants [35], whereas increased abundance of *Trichoderma* has been reported in Verticillium wilt–resistant cotton cultivars [36]. Consistent with these observations, distinct bacterial taxa were enriched in banana germplasms exhibiting different levels of *Fusarium* wilt resistance. Among the highly resistant germplasms, DJ and BDJ were characterized by a higher abundance of *Bacillus*. Members of this genus are widely recognized as beneficial rhizobacteria with antagonistic activity against a range of plant pathogens [37,38]. The enrichment of *Bacillus* in these germplasms suggests a potential association between this taxon and *Fusarium* wilt resistance. The moderately resistant germplasms DJ3H and HY5H were characterized by the enrichment of *Chujaibacter* and *Acinetobacter*, respectively. Previous studies have linked *Chujaibacter* to rhizosphere ecological processes and plant-associated environments [39]. Similarly, *Acinetobacter* is commonly found in plant rhizospheres and is involved in organic matter transformation and nutrient cycling [40,41,42]. Their enrichment in DJ3H and HY5H indicates that different banana germplasms may recruit distinct bacterial taxa with potentially important ecological functions. In the low-resistant germplasm DJ1H, *Burkholderia*–*Caballeronia*–*Paraburkholderia*, *Sphingomonas*, and *Rhodanobacter* were identified as enriched taxa. These bacterial groups have been reported to possess plant-associated functional traits, including nutrient acquisition, environmental adaptation, and plant growth promotion [43,44,45,46,47,48,49]. The occurrence of these taxa in DJ1H indicates that beneficial bacteria were also present in the rhizosphere of the low-resistant germplasm. Therefore, differences in *Fusarium* wilt resistance among banana germplasms may not depend solely on the presence or absence of individual beneficial taxa. Instead, they may be associated with variation in bacterial community composition and organization.

Previous studies have shown that beneficial rhizosphere microorganisms can suppress soil-borne pathogens by producing antibiotics, siderophores, and other secondary metabolites. Beneficial rhizosphere microorganisms can also limit pathogen establishment through resource competition and niche occupation [50]. These microbial activities are considered important components of disease suppression in the rhizosphere. In the present study, functional prediction analysis revealed differences in the potential metabolic profiles of rhizosphere bacterial communities among banana germplasms with different levels of *Fusarium* wilt resistance. The highly resistant germplasms DJ, BDJ, and NTH showed higher predicted abundances of pathways involved in carbohydrate metabolism, secondary metabolite biosynthesis, and antibiotic biosynthesis. These functional characteristics were generally consistent with the enrichment of potentially beneficial bacterial taxa in these germplasms and may be associated with their higher *Fusarium* wilt resistance [51,52]. It should be noted that PICRUSt2-based functional prediction reflects the potential functional capacity of microbial communities based on taxonomic composition rather than direct measurements of gene expression or metabolic activity [53]. Therefore, the predicted functional differences observed in this study should be interpreted with caution and require further experimental validation. The low-resistant germplasm DJ1H showed predicted abundances of pathways involved in secondary metabolite and antibiotic biosynthesis comparable to those of some highly resistant germplasms. Despite these similarities, DJ1H exhibited a much lower level of *Fusarium* wilt resistance. Notably, the predicted abundance of antibiotic biosynthesis pathways in DJ1H was similar to that in certain highly resistant germplasms. This may reflect community functional divergence that favors metabolites involved in intra-community competition rather than specific antagonism against Foc. Alternatively, this pattern may reflect differences in PICRUSt2 prediction accuracy among bacterial taxa and should be interpreted with caution. This result suggests that variation in *Fusarium* wilt resistance cannot be explained solely by differences in the abundance of individual predicted functional pathways. Instead, resistance may be associated with the combined influence of bacterial community composition, enrichment of specific functional taxa, and overall community organization.

Plant disease resistance is associated not only with the presence of specific beneficial microorganisms but also with the overall organization of microbial communities [54]. As rhizosphere microbial communities develop, complex association patterns can emerge among microbial taxa, potentially influencing community structure and ecological functioning [55,56,57]. Previous studies have reported that resistant cultivars of several crops, including grapevine and tobacco, often harbor more highly connected rhizosphere microbial networks than susceptible cultivars [58,59,60]. In addition, certain taxa occupying central positions within microbial networks have been suggested to play important roles in maintaining network organization and community functioning. These findings indicate that microbial interaction networks may provide additional insights into the ecological processes associated with plant disease resistance.

Previous studies have suggested that microbial association networks may provide insights into community organization and potential ecological relationships among microbial taxa [21,61,62,63]. In the present study, co-occurrence network analysis revealed distinct correlation patterns among bacterial taxa associated with banana germplasms exhibiting different levels of *Fusarium* wilt resistance. These correlations do not necessarily indicate direct ecological interactions. They may also arise from shared environmental preferences or other unmeasured ecological or methodological factors. Several taxa enriched in the highly resistant germplasms showed strong positive correlations with other bacterial groups. For example, MND1 showed significant positive correlations with Candidatus_Omnitrophus and *Pedomicrobium*, while *Gemmatimonas* was positively associated with unclassified_Acidobacteriales. In addition, *Terrimonas* exhibited positive associations with P3OB_42 and unclassified_TRA3_20. In contrast, bacterial taxa associated with moderately resistant and low-resistant germplasms exhibited diverse association patterns involving both positive and negative correlations. For example, *Chujaibacter*, enriched in DJ3H, showed significant negative correlations with multiple taxa. Although DJ1H was enriched in potentially beneficial taxa such as *Burkholderia*–*Caballeronia*–*Paraburkholderia*, the association patterns involving this taxon differed from those observed in highly resistant germplasms. When considered together with the community composition and functional prediction results, these findings indicate that banana germplasms with different levels of *Fusarium* wilt resistance harbor distinct bacterial community configurations. Differences in resistance may therefore be associated not only with the enrichment of specific bacterial taxa but also with variation in the overall patterns of bacterial associations within the rhizosphere community. Further studies integrating metagenomic, transcriptomic, or experimental approaches will be required to clarify the ecological significance of these observed association patterns.

Taken together, the results of this study demonstrate that banana germplasms differing in *Fusarium* wilt resistance harbor distinct rhizosphere bacterial communities. Differences in bacterial community composition, predicted functional profiles, and bacterial association patterns were observed among germplasms, highlighting the potential role of host genotype in shaping rhizosphere bacterial assemblages. This study was conducted in representative sandy loam soil from a banana-growing region of southern Yunnan under consistent water and fertilizer management. However, differences in soil texture, pH, and nutrient availability across regions may limit the generalizability of the findings. In addition, the ecological functions of many key taxa identified in this study remain unclear. Future studies integrating cultivation-based approaches, metagenomics, and functional validation experiments will help further clarify the roles of these bacteria in disease suppression and plant health.

## 5. Conclusions

This study aimed to investigate rhizosphere bacterial community characteristics associated with *Fusarium* wilt resistance in banana germplasms. Banana germplasms with different levels of *Fusarium* wilt resistance harbored distinct rhizosphere bacterial communities that differed in community composition, predicted functional profiles, and bacterial association patterns. The observed variation among germplasms suggests that host genotype plays an important role in shaping rhizosphere bacterial assemblages.

The results indicate that *Fusarium* wilt resistance in banana is associated with broader differences in rhizosphere bacterial community organization rather than bacterial diversity alone. These findings provide a basis for future studies investigating the ecological roles of disease-associated bacterial taxa and their potential applications in the microbiome-informed management of banana *Fusarium* wilt.

## Data Availability

All data generated or analyzed during this study are included in this published article.

## Supplementary Materials

The following supporting information can be downloaded at https://www.mdpi.com/article/10.3390/biology15141186/s1, Figure S1: ANOSIM analysis of differences in rhizosphere bacterial community structure among banana germplasms with different levels of resistance to *Fusarium* wilt; Table S1: Evaluation of *Fusarium* wilt resistance in banana germplasms; Table S2: Quality statistics of 16S rRNA sequencing data from rhizosphere bacterial communities of banana germplasms with different levels of resistance to *Fusarium* wilt; Table S3: Taxonomic annotation information of common OTUs; Table S4: Number of taxonomic units of rhizosphere bacterial communities at different taxonomic levels among banana germplasms with different levels of resistance to *Fusarium* wilt; Table S5: Alpha diversity indices of rhizosphere bacterial communities associated with banana germplasms exhibiting different levels of resistance to *Fusarium* wilt.
