# Supplementary material for: Rhizosphere Bacterial Community Structure and Functional Characteristics Associated with Fusarium Wilt Resistance in Banana Germplasms
Source: Biology (Basel). 2026 Jul 18;15(14):1186. doi: 10.3390/biology15141186 (PMC13403893; doi:10.3390/biology15141186)
Supplement: Supplementary file 1 [file biology-15-01186-s001.zip › Supplementary File/Tables.pdf]

Table S1. Evaluation of *Fusarium* wilt resistance in banana germplasms.

Banana germplasm	Composite disease severity index	Resistance grade	Resistance level
DJ1H	2.96±0.32	3	Low-resistance
HY5H	1.02±0.11	2	Moderately resistance
DJ3H	1.22±0.17	2	Moderately resistance
DJ4H	0.61±0.07	1	Highly resistance
NTH	0.43±0.05	1	Highly resistance
BDJ	0.49±0.04	1	Highly resistance
DJ	0.52±0.04	1	Highly resistance

Table S2. Quality statistics of 16S rRNA sequencing data from rhizosphere bacterial communities of banana germplasms with different levels of resistance to *Fusarium* wilt.

Sample ID	Raw CCS	Clean CCS	Effective CCS	AvgLen (bp)	Effective(%)
BDJ-1	60504	60473	58836	1451	97.24
BDJ-2	62204	62180	60308	1451	96.95
BDJ-3	55823	55798	53779	1450	96.34
BDJ-4	62183	62167	60112	1451	96.67
DJ-1	65448	65429	62853	1452	96.04
DJ-2	55336	55330	53221	1453	96.18
DJ-3	54833	54798	51577	1453	94.06
DJ-4	58182	58176	56406	1453	96.95
DJ1H-1	58439	58424	55814	1447	95.51
DJ1H-2	55798	55791	53748	1446	96.33
DJ1H-3	54432	54415	52635	1447	96.7
DJ1H-4	56554	56543	54223	1447	95.88
DJ3H-1	66568	66556	64340	1450	96.65
DJ3H-2	56696	56654	55554	1450	97.99
DJ3H-3	63830	63807	62619	1450	98.1
DJ3H-4	60567	60555	59151	1450	97.66
DJ4H-1	56415	56398	52404	1447	92.89
DJ4H-2	60258	60228	55923	1447	92.81
DJ4H-3	59947	59930	57812	1447	96.44
DJ4H-4	56390	56378	52600	1447	93.28
HY5H-1	59645	59621	54151	1451	90.79
HY5H-2	60199	60189	53792	1452	89.36
HY5H-3	63031	63018	56876	1451	90.23
HY5H-4	64363	64355	58433	1451	90.79
NTH-1	53814	53805	50922	1454	94.63
NTH-2	63254	63214	58913	1456	93.14
NTH-3	58091	58080	55510	1456	95.56
NTH-4	68188	68175	64919	1456	95.21

Table S4. Number of taxonomic units of rhizosphere bacterial communities at different taxonomic levels among banana germplasms with different levels of resistance to *Fusarium* wilt.

Sample	Kindom	Phylum	Class	Order	Family	Genus	Species
BDJ-1	2	34	73	219	418	839	1747
BDJ-2	2	31	74	220	418	849	1729
BDJ-3	2	33	74	218	417	816	1697
BDJ-4	2	31	73	209	422	827	1673
DJ-1	2	34	79	244	484	946	1887
DJ-2	2	35	82	249	484	933	1933
DJ-3	2	36	81	248	480	963	2073
DJ-4	2	34	77	237	456	888	1739
DJ1H-1	2	33	70	211	398	775	1585
DJ1H-2	2	32	70	201	377	761	1487
DJ1H-3	2	32	71	205	393	801	1635
DJ1H-4	2	31	69	204	389	789	1596
DJ3H-1	2	32	79	216	415	865	1785
DJ3H-2	1	31	75	209	402	836	1674
DJ3H-3	2	33	78	212	418	886	1844
DJ3H-4	1	31	76	217	427	887	1836
DJ4H-1	2	33	73	207	403	865	1771
DJ4H-2	2	32	75	213	421	894	1845
DJ4H-3	2	34	77	218	426	908	1892
DJ4H-4	2	33	74	210	418	887	1821
HY5H-1	2	34	76	219	427	896	2076
HY5H-2	2	34	78	219	420	854	1948
HY5H-3	2	33	74	217	427	883	2018
HY5H-4	2	35	78	220	416	877	1989
NTH-1	2	32	79	223	437	827	1596
NTH-2	2	34	82	243	475	982	2101
NTH-3	2	33	78	231	441	868	1778
NTH-4	2	33	80	241	467	930	1881
Total	2	37	92	307	679	1586	4281

Table S5. Alpha diversity indices of rhizosphere bacterial communities associated with banana germplasms exhibiting different levels of resistance to *Fusarium* wilt.

Sample ID	Feature	ACE	Chao1	Simpson	Shannon	PD_whole_tree	Coverage
BDJ-1	8396	12621.6943	12346.3785	0.9985	11.3323	32.8165	0.9383
BDJ-2	8619	12852.8166	12444.8194	0.9985	11.3884	32.2861	0.9383
BDJ-3	8313	15415.4212	12728.4619	0.9986	11.3855	32.7712	0.9268
BDJ-4	8550	12775.3104	12578.9449	0.9986	11.3749	32.6573	0.9385
DJ-1	8564	12122.9801	11873.1492	0.9991	11.5432	27.4876	0.9458
DJ-2	8235	12053.0982	11592.3346	0.9991	11.5507	27.4744	0.9352
DJ-3	8535	13133.5737	12712.0959	0.999	11.5844	28.6423	0.9241
DJ-4	8003	11509.5228	11416.0037	0.999	11.456	27.0777	0.9435
DJ1H-1	7643	13353.0249	11106.3139	0.9984	11.0833	32.1634	0.9413
DJ1H-2	7355	10886.53	10657.7865	0.9984	11.0711	31.2681	0.9422
DJ1H-3	7695	13792.0894	11363.0819	0.9985	11.1614	32.0909	0.9338
DJ1H-4	7687	13872.5399	11479.2406	0.9985	11.1205	32.9646	0.9366
DJ3H-1	6016	10901.2201	8950.1836	0.9895	9.6144	30.7251	0.9605
DJ3H-2	5645	10201.3429	8581.308	0.9906	9.6534	30.8558	0.9555
DJ3H-3	6054	11323.0647	9151.8669	0.99	9.6238	30.8026	0.9575
DJ3H-4	6055	11684.5863	9271.9491	0.9909	9.7267	30.3296	0.954
DJ4H-1	6250	12481.015	10018.0176	0.9927	10.129	32.8281	0.9435
DJ4H-2	6533	12401.0108	10156.7008	0.9938	10.2525	33.5472	0.9461
DJ4H-3	6667	12668.8929	10370.0438	0.9931	10.208	33.3312	0.9472
DJ4H-4	6511	13130.6079	10469.4732	0.9934	10.2431	33.1126	0.9404
HY5H-1	7209	16206.0956	12185.7003	0.9911	9.9589	32.1488	0.9253
HY5H-2	6623	13880.7353	10730.2786	0.9892	9.7396	32.8722	0.935
HY5H-3	7151	14128.8292	11244.8656	0.9907	9.9182	33.2561	0.9369
HY5H-4	7207	14837.5122	11618.1558	0.9907	9.9362	33.2078	0.9363
NTH-1	7107	10474.8215	10346.0247	0.9983	11.0821	28.0303	0.9421
NTH-2	8519	15468.233	12726.1513	0.9984	11.305	29.501	0.9329
NTH-3	7631	11409.3504	11379.0373	0.9983	11.1546	29.3872	0.9411
NTH-4	8273	12337.9956	12279.4503	0.9984	11.2038	30.1619	0.9454
