# Supplementary figures and images for: Rhizosphere Bacterial Community Structure and Functional Characteristics Associated with Fusarium Wilt Resistance in Banana Germplasms
Source: Biology (Basel). 2026 Jul 18;15(14):1186. doi: 10.3390/biology15141186 (PMC13403893; doi:10.3390/biology15141186)

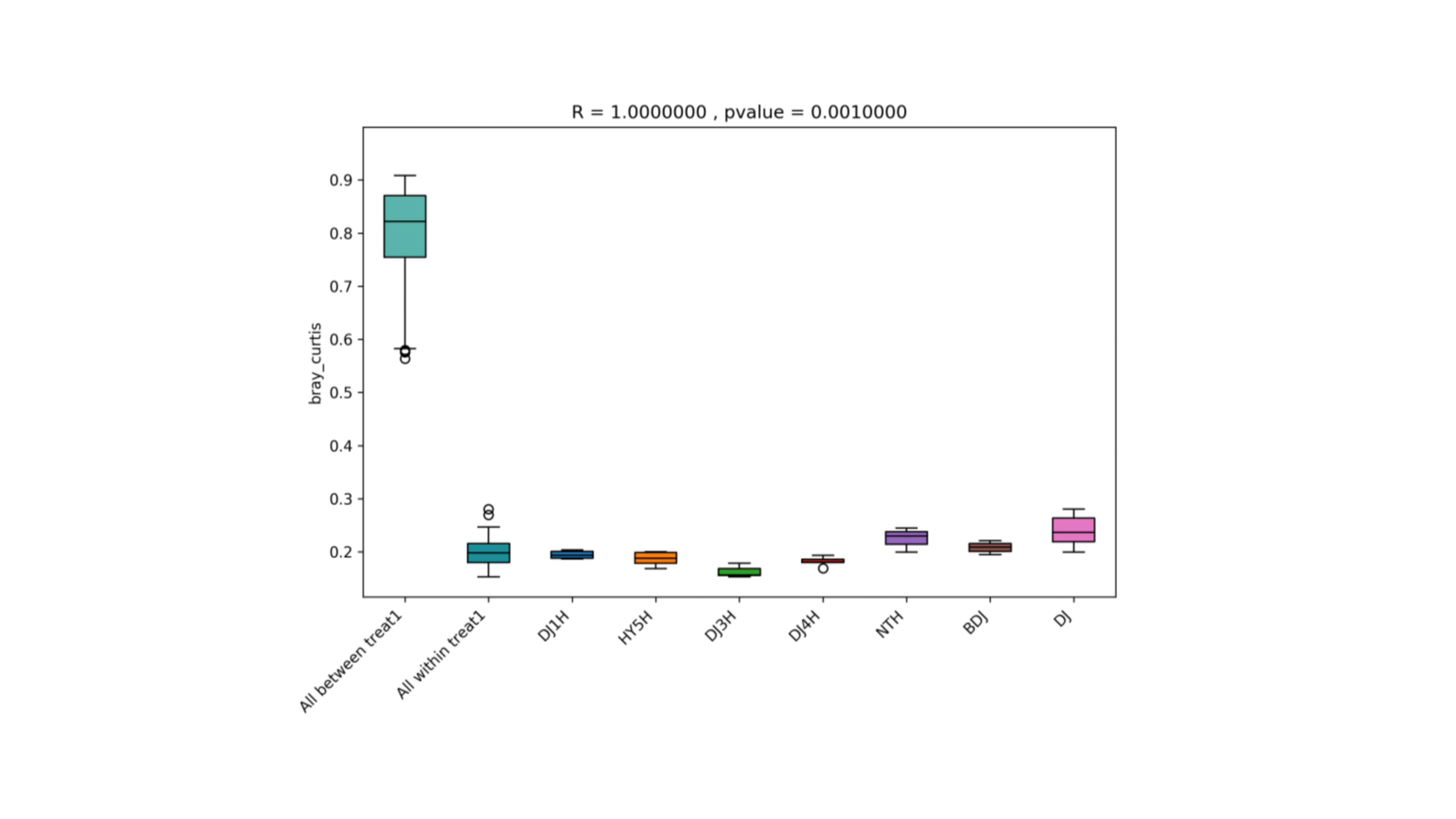

Supplement: Supplementary file 1 [file biology-15-01186-s001.zip › Supplementary File/Figure S1.png]
